# Zearalenone Inhibits Rat and Human 11*β*-Hydroxysteroid Dehydrogenase Type 2

**DOI:** 10.1155/2015/283530

**Published:** 2015-12-20

**Authors:** Linxi Li, Xiaolong Wu, Hongguo Guan, Baiping Mao, Huang Wang, Xiaohuan Yuan, Yanhui Chu, Jianliang Sun, Ren-Shan Ge

**Affiliations:** ^1^Center of Scientific Research, The Second Affiliated Hospital & Yuying Children's Hospital of Wenzhou Medical University, Wenzhou, Zhejiang 325027, China; ^2^Department of Anesthesiology, The Second Affiliated Hospital & Yuying Children's Hospital of Wenzhou Medical University, Wenzhou, Zhejiang 325027, China; ^3^Heilongjiang Key Laboratory of Anti-Fibrosis Biotherapy, Mudanjiang Medical University, Heilongjiang 157011, China; ^4^Department of Anesthesia, Hangzhou Hospital Affiliated to Nanjing Medical University, Hangzhou First People's Hospital, Hangzhou 310006, China

## Abstract

Zearalenone is a mycotoxin produced by* Fusarium* spp. 11*β*-Hydroxysteroid dehydrogenases, isoforms 1 (HSD11B1) and 2 (HSD11B2), have been demonstrated to be the regulators of the local level of active glucocorticoid, which has a broad range of physiological actions. In the present study, the potency of zearalenone was tested for the inhibition of HSD11B1 and HSD11B2 in rat and human tissues. Zearalenone showed potent inhibition of HSD11B2 with the half-maximal inhibitory concentration (IC_50_) calculated at 49.63 and 32.22 *μ*M for the rat and human, respectively. Results showed that zearalenone competitively inhibited HSD11B2 when a steroid substrate was used. However, it served as an uncompetitive inhibitory factor when the cofactor NAD^+^ was used. In contrast, the potency of zearalenone to inhibit both rat and human HSD11B1 was diminished, with the concentration of 100 *μ*M causing almost no inhibitory effect on the isoform. In conclusion, we observed that zearalenone is a selective inhibitor of HSD11B2, implying that this agent may cause excessive glucocorticoid action in local tissues such as kidney and placentas.

## 1. Introduction

Zearalenone is a mycotoxin produced by* Fusarium* spp. [1]. Although zearalenone is structurally dissimilar to estradiol, it possesses estrogenic activity. The molecule of zearalenone undergoes folding such that the molecule can bind to an estrogen receptor [1]. Zearalenone is found in a number of cereal crops, such as wheat, maize, barley, oats, rice, and sorghum [2]. It is very stable in foods even during heat processing. Therefore, it can be ingested into human bodies without damage of its active molecule.

Zearalenone mainly targets reproductive system [3], because it has weak estrogen activity. Apparently, it has about 20-fold lower binding to estrogen receptor than the endogenous estrogen estradiol [1]. However, zearalenone has also been shown to exert toxicity to immune system, liver, and lipid peroxidation [4].

**Figure 1:**
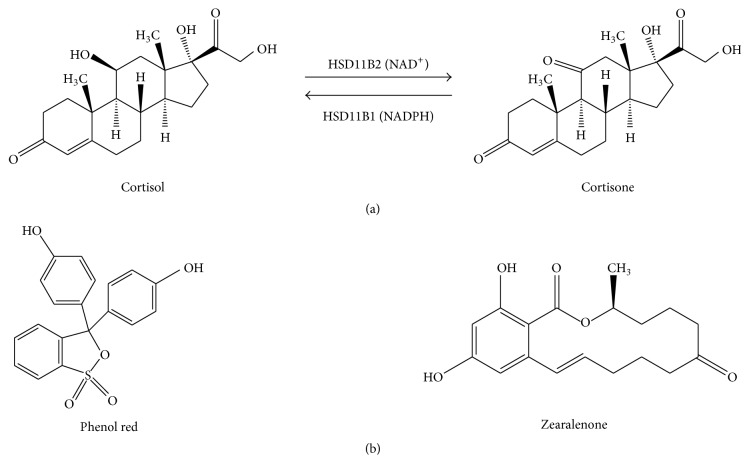
The illustration of catalytic reactions of 11*β*-hydroxysteroid dehydrogenase isoforms (HSD11B1 and HSD11B2) (a) and the chemical structure of phenol red and zearalenone (b).

Glucocorticoids play a wide range of physiological and pharmacological roles in mammalian physiology [5, 6]. Glucocorticoid exerts its actions after binding to its nuclear receptor, glucocorticoid receptor, thus regulating gene expression in target tissues and thereby causing their action. Intracellular levels of glucocorticoids (cortisol in humans and corticosterone in rats) are controlled by the glucocorticoid metabolizing enzyme 11*β*-hydroxysteroid dehydrogenase (HSD11B). There are two known isoforms. Type I isoform, HSD11B1, is NADP^+^/NADPH dependent oxidoreductase that catalyzes the interconversion of 11*β*-hydroxyl steroids and 11-keto steroids, for example, cortisone in humans and 11-dehydrocorticosterone (11DHC) in rats. The HSD11B1 isoform is most abundantly expressed in glucocorticoid target tissues such as liver, fat, brain, and testis [7] (Figure 1). HSD11B1 has low-affinity binding to its steroid substrate with *K*_*m*_ of approximately 2 *μ*M [8]. HSD11B1 behaves predominantly as a reductase in liver and fat, thus regenerating active 11*β*-hydroxyl glucocorticoids from the circulatory inactive 11-keto glucocorticoids [7]. Interestingly, increased HSD11B1 activity in the liver and adipose tissue has been thought to contribute to the etiology of metabolic syndromes [7].

The second isoform, HSD11B2, is a unidirectional NAD^+^ dependent oxidase, which inactivates 11*β*-hydroxyl glucocorticoids (Figure 1), thereby preventing binding of the mineralocorticoid receptor by natural glucocorticoids [9]. Genetically null mutations of the* HSD11B2* gene in the human cause a syndrome named apparent mineralocorticoid excess in which circulatory aldosterone levels are subnormal and are related to hypertension and hypokalemia [10]. Furthermore, HSD11B2 is thought to be a critical gatekeeper that eliminates active glucocorticoids in the maternal side of the placenta in order to protect the fetus from unusually high glucocorticoid concentrations [11]. Indeed, a previous report demonstrated that mutation and inhibition of HSD11B2 that was occasioned by exposure to chemicals caused placental dysfunction associated with low birth weight [12]. By virtual screening, we have observed that zearalenone is a possible inhibitor of the HSD11B enzyme. In the present study, we performed experiments to determine the direct suppression of both HSD11B isoform and the possible mechanism of zearalenone. Our findings suggest that zearalenone is primarily a selective inhibitor of HSD11B2 in rat and human tissues.

## 2. Materials and Methods

### 2.1. Chemicals and Animals

[1,2-^3^H]-Corticosterone (^3^H-corticosterone) (specific activity: 40 Ci/mmol) and [1,2,6,7-^3^H(N)]-cortisol (specific activity: 70–100 Ci/mmol) were purchased from DuPont-New England Nuclear (Boston, MA). [1,2-^3^H]-11-Dehydrocorticosterone (^3^H-11DHC) and [1,2,6,7-^3^H(N)]-cortisone were prepared from labeled ^3^H-corticosterone or ^3^H-cortisol as described earlier [13]. Unlabeled corticosterone, 11DHC, cortisol, and cortisone were purchased from Steraloids (Wilton, NH). Zearalenone was purchased from Sigma (St. Louis, MO). Male Sprague-Dawley rats (250–300 g) were purchased from Shanghai Laboratory Animal Center (Shanghai, China). The experimental protocol was approved by the Wenzhou Medical University's Animal Care and Use Committee. Human liver microsomes were purchased from BD Gentest (NJ, USA). Full-term human placentas were obtained from the 2nd Affiliated Hospital of Wenzhou Medical University under the approval of the Ethics Committee of the hospital.

### 2.2. Preparation of Microsomal Protein

Microsomal preparations of rat liver and kidney and human placentas were prepared as described previously [14]. Briefly, samples were homogenized in cold 0.01 M phosphate buffered saline (PBS) containing 0.25 M sucrose and centrifuged at 700 ×g for 30 min. The supernatants were transferred to new tubes and centrifuged at 10,000 ×g for 30 min. The supernatants were centrifuged twice at 105,000 ×g for 1 hour (twice). Pellets were resuspended and protein contents were measured. The protein concentrations were measured using the Bio-Rad Protein Assay Kit (cat.# 500-0006, Bio-Rad, Hercules, CA) according to the manufacturer's protocol. Microsomes from all tissues were used for measurement of HSD11B activities.

### 2.3. HSD11B Assay

HSD11B1 activity was measured in rat and human liver microsomes using [^3^H]-11DHC or [^3^H]-cortisone, respectively, according to a previously described method [14]. The assay tubes contained 200 nM cortisone or 11DHC and 0.2 mM NADPH and 2 mM glucose-6-phosphate. The microsomes were preincubated with zearalenone for 2 min and then added to the above tubes. The initial test concentration for zearalenone was 100 *μ*M.

HSD11B2 activity was measured in rat kidney and human placental microsomes using [^3^H]-corticosterone or [^3^H]-cortisol, respectively, according to a previously described method [14]. The assay tubes contained 25 nM corticosterone or cortisol and 0.2 mM NAD^+^ and 2 mM DTT. The microsomes were preincubated with zearalenone for 2 min and then added to the above tubes. The initial test concentration for zearalenone was 100 *μ*M. When the inhibitory concentration was established, different concentrations of zearalenone were used.

On each occasion, the reactions were stopped by adding 2 mL ice-cold ether. The steroids were extracted, and the organic layer was dried under nitrogen. The steroids were separated chromatographically on thin layer plates in chloroform and methanol (90 : 10, v/v), and the radioactivity was measured using a scanning radiometer (System AR2000, Bioscan Inc., Washington, DC). HSD11B1 activity was determined by the percentage conversion of 11DHC to corticosterone or cortisone to cortisol and HSD11B2 activity by the conversion of corticosterone to 11DHC or cortisol to cortisone by dividing the radioactive counts identified by the total counts associated with substrate and products.

### 2.4. Analysis of Enzyme Kinetics

The dose-dependent inhibition of zearalenone on HSD11B was subjected to nonlinear analysis by GraphPad (Version 6, GraphPad Software Inc., San Diego, CA), and half-maximal inhibitory concentrations (IC_50_) were calculated. Michaelis-Menten kinetics and Lineweaver-Burk plots were drawn for the analysis of the mode of inhibition of zearalenone.

### 2.5. Statistics

Each experiment was repeated four to six times. Data were subjected to analysis by unpaired Student *t*-tests to identify significant differences between two groups. All data are expressed as means SEM. Differences were regarded as significant at *P* < 0.05.

## 3. Results

### 3.1. Effects of Zearalenone on HSD11B1 Activity

**Figure 2:**
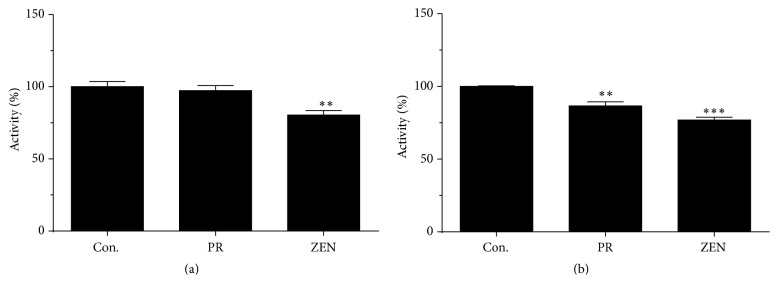
The effects of zearalenone (ZEN) and phenol red (PR) on rat (a) and human 11*β*-hydroxysteroid dehydrogenase isoform 1 (HSD11B1) (b) were tested at the 100 *μ*M concentration. Graph includes results from four to six separate and independent experiments. Mean ± SEM; *∗∗* and *∗∗∗* indicate significant difference compared to control at *P* < 0.01 and 0.001, respectively.

**Figure 3:**
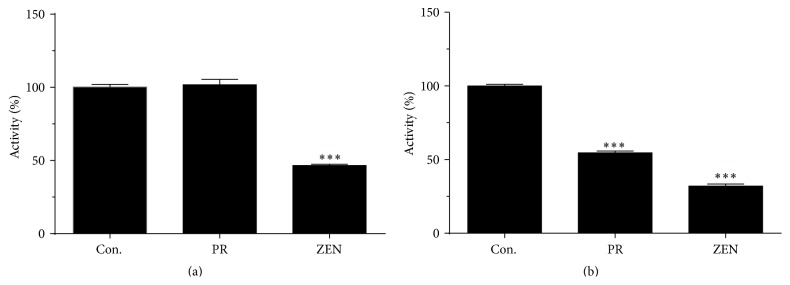
The effects of zearalenone (ZEN) and phenol red (PR) on rat (a) and human 11*β*-hydroxysteroid dehydrogenase isoform 2 (HSD11B2) (b) were tested at the 100 *μ*M concentration. Graph includes results from four to six separate and independent experiments. Mean ± SEM; *∗∗∗* indicates significant difference compared to control at *P* < 0.001.

Because HSD11B1 is an oxidoreductase, reductase activity was measured in the presence of the cofactor NADPH. We also compared the zearalenone with another weak estrogen phenol red, which has also hydroxyl group in its chemical structure. At 100 *μ*M, zearalenone showed weak but significant inhibition of the rat HSD11B1 reductase (Figure 2(a)), while phenol red did not affect rat HSD11B1 activity (Figure 3(b)). Both zearalenone and phenol red weakly inhibited human HSD11B1 activity (Figure 2(b)). However, the inhibition of HSD11B1 by 100 *μ*M zearalenone never exceeded 50%. Together, results indicate that both phenol red and zearalenone have almost no inhibitory effect on both rat and human HSD11B1 at 100 *μ*M or below.

### 3.2. Effects of Zearalenone on HSD11B2 Activity

**Figure 4:**
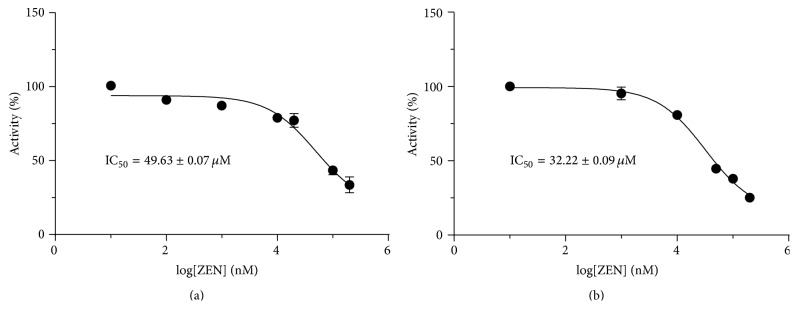
Dose-dependent inhibition of rat (a) and human 11*β*-hydroxysteroid dehydrogenase isoform 2 (HSD11B2) by zearalenone (ZEN) (b). Mean ± SEM; graph includes results from four separate and independent experiments.

HSD11B2 is a unidirectional oxidase, and its activity was measured in the presence of the cofactor NAD^+^. Zearalenone exhibited potent and significant inhibition (>50%) of rat and human HSD11B2 activity at the 100 *μ*M concentration (Figures 3(a) and 3(b)). Phenol red had no inhibition on rat HSD11B2 activity, but it inhibited human HSD11B2 activity (Figure 3). In addition, analysis of dose-dependent zearalenone inhibition of HSD11B2 activity estimated IC_50_ values of 49.63 ± 0.07 and 32.22 ± 0.09 *μ*M for rat and human HSD11B2 activities, respectively (Figure 4). The results imply that zearalenone has a potent to moderate capacity to cause inhibition of both rat and human HSD11B2.

### 3.3. The Mode of Inhibition of Zearalenone on HSD11B2 Activity

**Figure 5:**
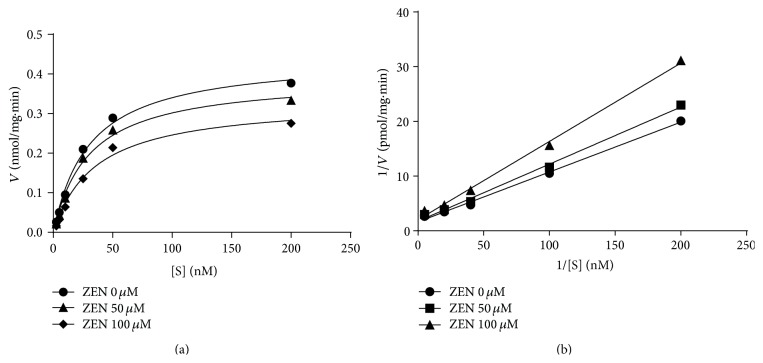
The mode of action of zearalenone (ZEN) on human 11*β*-hydroxysteroid dehydrogenase isoform 2 (HSD11B2). The HSD11B2 activity was analyzed using different concentrations of cortisol. (a) Michaelis-Menten kinetics; (b) Lineweaver-Burk plots. Graph includes results from four separate and independent experiments.

**Figure 6:**
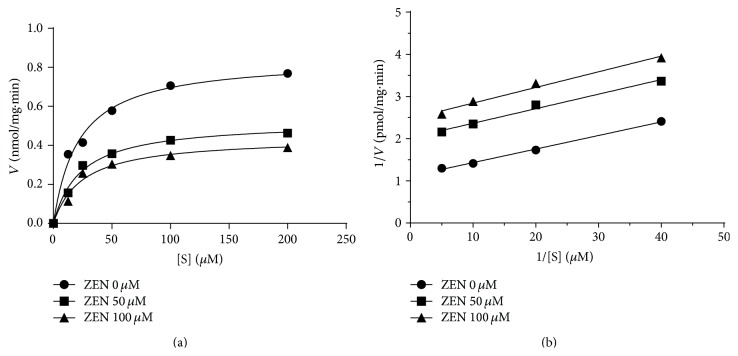
The mode of action of butylated hydroxyanisole (ZEN) on human 11*β*-hydroxysteroid dehydrogenase isoform 2 (HSD11B2). The HSD11B2 activity was investigated using different concentrations of cofactor NAD^+^. (a) Michaelis-Menten kinetics; (b) Lineweaver-Burk plots. Graph includes results from four separate and independent experiments.

The HSD11B2 enzyme has dual substrates, that is, steroid and cofactor (NAD^+^). At first, we tested the mode of inhibition of zearalenone on this enzyme when cortisol was used for human enzyme. Michaelis-Menten kinetics (Figure 5(a)) and Lineweaver-Burk plots (Figure 5(b)) showed that zearalenone was a competitive inhibitor for human HSD11B2. This was true for rat HSD11B2 when corticosterone was used (data not shown). This indicates that zearalenone competes with the steroid substrate at its binding site. When the cofactor NAD^+^ was used, Michaelis-Menten kinetics (Figure 6(a)) and Lineweaver-Burk plots (Figure 6(b)) showed that zearalenone was an uncompetitive inhibitor for human HSD11B2. This was also true for rat HSD11B2 when corticosterone was used (data not shown). These results indicate that zearalenone binds to HSD11B2-NAD^+^-corticosterone complex.

## 4. Discussion

The widespread availability of zearalenone from the food resources has given rise to public concerns about its adverse effects on the population. Although many studies have investigated the pharmacological activities of zearalenone in several animal models for its toxicity in reproductive system, the mechanisms of action of zearalenone are not fully understood. The present study showed that zearalenone inhibited the HSD11B2 enzyme in rat and human tissues with IC_50_ values in the range 32–49 *μ*M. It virtually has almost no inhibitory effect on HSD11B1 isoform. This finding indicates that zearalenone is a more specific inhibitor of the HSD11B2 enzyme.

In the placentae, HSD11B2 protects the fetus from the high circulating levels of maternal glucocorticoids [11, 15, 16] by helping in elimination of maternal cortisol [17, 18]. Mouse models with null mutation of* Hsd11b2* gene showed placental anomalies and dysfunction that resulted in intrauterine fetal growth retardation [12]. Thus, our observations imply that zearalenone-related inhibition of HSD11B2 during pregnancy is associated with glucocorticoid-mediated effects that potentially cause adverse consequences on fetal development.

Moreover, HSD11B2 is thought to be the gatekeeper for mineralocorticoid receptors in its target tissue such as kidney and colon [10]. Usually, mineralocorticoid receptors have no selectivity for aldosterone (a natural mineralocorticoid) or cortisol (a natural glucocorticoid) with serum concentrations that are 100-fold greater than aldosterone [19]. Thus, the high affinity of HSD11B2 for cortisol effectively inactivates this compound through its metabolic conversion to cortisone, which has negligible binding affinity to the mineralocorticoid receptor [20, 21]. Indeed, null mutation of the* HSD11B2* [22] or pharmacological inhibition by chemicals has shown that mineralocorticoid receptors in the kidney are occupied by cortisol, causing apparent mineralocorticoid excess associated in part with hypertension and hypokalemia [23, 24]. It does appear that the HSD11B2 isoform is essential for maintaining physiological levels of aldosterone activity.

There is evidence that zearalenone may interfere with development of the testis and male reproductive tract. Mice administered orally with a single dose (zearalenone) had reduced testosterone level [25]. Adult male mice were administered intraperitoneally with zearalenone at 0, 25, 50, and 75 mg/kg body weight daily for 7 days, and zearalenone significantly reduced serum testosterone level [26]. HSD11B2 has been shown to be present in fetal and adult mammalian (human, rat, and pig) Leydig cells [27–29]. Therefore, HSD11B2 may play an important role in glucocorticoid inactivation in Leydig cells to prevent glucocorticoid-mediated suppression of testosterone production, because glucocorticoid cortisol or corticosterone directly binds to the glucocorticoid receptor in Leydig cells and inhibits androgen biosynthesis [29–31].

Overall, zearalenone has greater selectivity for HSD11B2 versus the HSD11B1 enzyme, because it inhibited rat and human HSD11B2 with IC_50_ values of 32–49 *μ*M, while it almost lacked effective inhibition of rat and human HSD11B1 even at 100 *μ*M. Structurally, zearalenone is a phenol with a hydroxyl group, which is similar to 11-beta hydroxyl group in cortisol and cortisone. The action of zearalenone on HSD11B2 appears to be similar to that reported for another polyphenolic compound, that is, gossypol, which is also highly selective for HSD11B2 compared to HSD11B1 [14]. However, HSD11B2 is a dual substrate enzyme, which needs the cofactor NAD^+^. When NAD^+^ was used, zearalenone showed mixed uncompetitive inhibition, indicating that zearalenone binds to NAD^+^-bounded enzyme complex.

## 5. Conclusions

We tested the effects of zearalenone on HSD11B1 and HSD11B2 and found that zearalenone selectively inhibited both rat and human HSD11B2 enzyme. The mode of action of zearalenone on HSD11B2 is a competition when a glucocorticoid substrate is used.

## Abbreviations

11DHC: 11-Dehydrocorticosterone
HSD11B: 11*β*-Hydroxysteroid dehydrogenase
HSD11B1: 11*β*-Hydroxysteroid dehydrogenase 1
HSD11B2: 11*β*-Hydroxysteroid dehydrogenase 2
IC_50_: Half-maximal inhibitory concentrations
PBS: Phosphate buffered saline
PR: Phenol red
ZEN: Zearalenone.
